# Knowledge, attitude, and practice regarding menopause management among primary healthcare providers in Kelantan, Malaysia: A cross-sectional study

**DOI:** 10.51866/oa.26-00003

**Published:** 2026-10-29

**Authors:** Nur Humairah Che Ibrahim, Ying Ying Ng, Juliawati Muhammad, Nani Draman

**Affiliations:** 1 Department of Family Medicine, School of Medical Sciences, Universiti Sains Malaysia, Kubang Kerian, Kelantan, Malaysia.

**Keywords:** Health knowledge, attitudes, practice, Menopause, Primary

## Abstract

**Introduction:**

Menopause marks the end of a woman’s reproductive years. Primary healthcare providers (PCPs) play a pivotal role in identifying menopausal symptoms, providing counselling and initiating management. However, information on their knowledge, attitudes and clinical practices in Malaysia remains limited. This study aimed to assess the knowledge, attitudes and practices regarding menopause management among PCPs in Kelantan.

**Methods:**

A cross-sectional study was conducted among PCPs in Kelantan health clinics using a self-administered questionnaire adapted from the Malaysian Clinical Practice Guidelines (CPGs) on Menopause Management. Simple and multiple linear regression analyses were used to identify factors associated with knowledge scores.

**Results:**

A total of 212 PCPs participated. The mean knowledge score was 19.45 (standard deviation [SD]=4.90) out of 30 (64.8%), while the mean attitude score was 38.97 (SD=3.83) out of 50 (72.4%). Among the participants, 42.0% often or always advised lifestyle modifications, whereas 52.8% had never prescribed menopausal hormone therapy (MHT) or referred women with menopausal symptoms for MHT. Higher knowledge scores were associated with older age (adjusted β=0.21; 95% confidence interval [CI]=0.08–0.34; P=0.002), family medicine specialist (FMS) designation (adjusted β=3.06; 95% CI=0.26–5.81; P=0.032) and use of CPGs as an information source (adjusted β=1.91; 95% CI=0.30–3.52; P=0.021).

**Conclusion:**

The PCPs demonstrated moderate knowledge of several menopause-related concepts and favourable attitudes. Higher knowledge scores were associated with older age, FMS designation and CPG use. Targeted education and guideline support may strengthen menopause management in primary care.

## Introduction

Natural menopause is defined as the permanent cessation of menstruation for 12 consecutive months, marking the end of a woman’s reproductive age. In Malaysia, the average age of menopause is 50.7 years, although a local study from Kota Bharu, Kelantan, found a slightly lower mean age of 49.4±3.4 years.^1,2^ With increasing female life expectancy now reaching 77.4 years, women are expected to spend a substantial proportion of their lives in the postmenopausal phase, making the management of menopausal health an increasingly important public health priority. However, menopause is often perceived as a natural life transition, particularly in conservative and predominantly Muslim communities, which may reduce healthcare utilisation despite distressing symptoms, leading many women to remain untreated or rely on alternative remedies.^2,3^

Menopause is frequently accompanied by a wide range of symptoms that can significantly impair quality of life. A study in Kelantan highlighted that local women experience significant symptoms, with somatic complaints (tiredness [79.1%] and musculoskeletal aches [70.6%]) being the most prevalent.^1^ Furthermore, declining sex steroid hormone levels increase vulnerability to chronic non- communicable diseases, including cardiovascular disease and osteoporosis. Malaysia is projected to experience the fastest growth in hip fractures among Asian countries.^4^

Menopausal hormone therapy (MHT) is the recommended first-line treatment for moderate-to- severe symptoms in healthy women under 60 years old.^5^ In addition to symptom relief, MHT has been shown to improve quality of life and may confer protective effects against osteoporosis and cardiometabolic conditions when initiated appropriately. However, despite these benefits and the availability of national clinical practice guidelines (CPGs), MHT utilisation in Malaysia remains low, with reported usage as low as 8.1%.^6^

Previous studies have suggested that the treatment gap in menopause care may be associated with doctor-, system- and patient-related factors. A recent study in the Klang Valley showed that although a large proportion of primary care doctors discussed MHT with patients (83.5%), the actual prescription rate was substantially low (0.9%), with most providers opting to refer patients to secondary care for initiation.^7^ Key barriers reported included 90.2% of PCPs lacking experience in prescribing MHT and 83% reporting no related training in the past year. These findings reflect a broader global pattern of ‘prescription inertia’, where positive attitudes towards MHT do not necessarily translate into clinical practice.

The knowledge—attitude—practice framework provides a structure for examining knowledge, attitudes and reported clinical practices. While studies have examined menopause management in urban settings, evidence from semi-urban and rural areas such as Kelantan remains limited. As primary healthcare providers (PCPs) are often the first point of contact, understanding their competence and practice is essential to improve care and patient outcomes.

Therefore, this study aimed to assess knowledge, attitudes and practices regarding menopause management among PCPs in Kelantan and identify factors associated with knowledge levels. By identifying gaps in knowledge, attitudes and self-reported practices, together with factors associated with knowledge, this study may help inform targeted educational and system-level interventions to strengthen menopause management in Malaysian primary care settings.

## Methods

### Study design

This study used a cross-sectional design to assess knowledge, attitudes and practices towards menopause management among PCPs working in public health clinics in Kelantan, Malaysia. It was conducted from September to December 2024 and was reported in accordance with the STROBE guidelines for cross-sectional studies.

### Study population and sampling

The study population included medical officers and family medicine specialists (FMSs) currently practising in public primary care clinics in Kelantan. House officers and medical officers with less than 6 months of working experience in health clinics were excluded from the study. A convenience sampling method was employed in this study.

### Data measurement tools

A questionnaire consisting of four sections was utilised. The first section collected data on sociodemographic and job characteristics including age, sex, ethnicity, job designation, place and years of practice, previous menopause-related training, gynaecology experience, personal or family history of receiving MHT and number of female patients aged above 40 years encountered per week.

The second section evaluated knowledge of menopause management. This section included a questionnaire developed and adapted from the Malaysian CPGs on Menopause Management, published in 2022. The questionnaire comprised nine domains: definition of menopause (Q1–2), symptoms of menopause (Q3–7), risk and complication of menopause (Q8–9), investigation to be conducted among perimenopausal women (Q10–11), indication for MHT (Q12–14), contraindication for MHT (Q15–18), initiation and continuation of MHT (Q19–24), side effects of MHT (Q25–27) and possible causes of non-response to MHT (Q28–30). Each item had three answer options: ‘true’, ‘false’ and ‘unsure’. For scoring, each correct answer was scored 1, while incorrect and ‘unsure’ answers were scored 0. The scores for each item were summed, yielding a total score ranging from 0 to 30. Items 2, 9, 11, 14, 15, 19, 21 and 24 were phrased as false statements.

The third section assessed attitudes towards menopause management. This section consisted of 10 items, which were scored using a 5-point Likert scale, ranging from 1 (strongly disagree) to 5 (strongly agree). Items 6 and 8 were negatively worded and reverse-scored to ensure directional consistency and reduce acquiescence bias. Total possible scores for this section ranged from 10 to 50. Higher total scores indicated a more favourable attitude towards managing menopause.

The fourth section comprised 10 items assessing the practice of menopause management. Responses were rated on a 5-point frequency scale. Practice responses were analysed at the individual-item level using frequencies and percentages, with interpretation focused on activities relevant to the Malaysian CPGs.

Total knowledge and attitude scores were summarised using means and standard deviations (SDs). Corresponding percentage scores were calculated to express the knowledge and attitude results on a 0–100 scale.

For the knowledge domain, the minimum possible score was 0. Therefore, percentage scores were calculated by dividing the total score by the maximum possible score of 30 and multiplying by 100. For the attitude domain, which had a theoretical score range of 10–50, the mean percentage score was calculated using the formula recommended by Hasson and Arnetz^8^:


$$\text{Mean Percentage}=\frac{\text{Mean Score}-\text{minimum score}}{\text{Maximum Score}-\text{minimum score}}\times 100$$

The questionnaire underwent content and face validation. An expert panel comprising four FMSs and two gynaecologists reviewed the items for clarity, relevance and alignment with the current national CPGs. Face validity was assessed among 10 medical officers to ensure clarity and comprehensibility. Subsequently, a pilot study was conducted among 65 PCPs at Klinik Rawatan Keluarga, Hospital Pakar Universiti Sains Malaysia. Although administratively located within a university hospital, the clinic functions as a primary care facility under the Department of Family Medicine and provides general outpatient and non-communicable disease care. It also manages non-pregnant women across different age groups, including women in the perimenopausal and postmenopausal age ranges. The pilot site was selected because its primary care functions and exposure to women’s health were broadly comparable to those of the target providers. Its staff were separate from the public health clinic providers recruited for the main study, thereby avoiding overlap between the pilot and main study samples and reducing the possibility that prior exposure to the questionnaire could influence responses in the main study.

During pilot testing, the questionnaire sections demonstrated acceptable-to-good internal consistency, with Cronbach’s alpha values of 0.717 for the knowledge section and 0.882 for both the attitude and practice sections.

### Sample size calculation

The sample size was calculated based on the mean attitude score using an online sample size calculator (http://wnarifin.github.io).^9^ Based on an SD of 6.7 from a pilot study and a precision of 1.0, a minimum of 173 participants were required. After a 20% non-response rate was accounted for, the final target sample size was 217.

### Data collection procedure

Data were collected through the online questionnaire (Google Forms) using convenience sampling. The survey link was disseminated through the official communication channels (WhatsApp groups) of the Kelantan Family Medicine Specialists Association, which then cascaded the invitation to medical officers under their supervision. Because the invitation was cascaded through these professional networks, the exact number of eligible providers who received or viewed the survey could not be determined. Consequently, a response rate could not be calculated, and information on non-responders was unavailable. All questionnaire items were set as mandatory in the online form; therefore, only complete submissions were recorded, and there were no missing data. Participation was voluntary, and informed consent was obtained electronically on the first page of the survey before data collection. Responses were collected anonymously, and no personally identifiable information was recorded.

### Data analysis

Data were analysed using SPSS 28 (IBM Corp., Armonk, New York, United States of America). Descriptive statistics including frequencies, percentages, means and SDs were used to summarise participant characteristics and knowledge and attitude scores. Simple linear regression (SLR) was initially performed to identify factors associated with knowledge scores. Independent variables included sociodemographic factors (age, sex, race and history of MHT) and job-related factors (designation, clinic type with or without an FMS, years of practice, gynaecology experience, training history and CPG usage). Variables with a P-value of less than 0.25 in the univariable analysis were considered for inclusion in the multiple linear regression (MLR) model based on the purposeful variable selection approach.^10^ MLR was utilised to identify significant independent predictors of knowledge scores while controlling for potential confounders. Multicollinearity was checked to ensure the model’s validity. Statistical significance for the final model was set at P<0.05.

### Ethical considerations

Ethical approval was obtained from the Human Research Ethics Committee of Universiti Sains Malaysia (USM/JEPeM/KK/24040336) and the Medical Research and Ethics Committee of the Ministry of Health Malaysia (NMRR ID-24-01739-UYI). Permission to conduct the study was also obtained from the Kelantan State Health Department. All data were handled confidentially and used solely for research purposes.

## Results

### Sociodemographic and job characteristics

A total of 212 PCPs participated in the study. The majority of the participants were women (84.4%) and worked as medical officers (93.4%). The mean age was 35.6 years (SD=5.3), and the participants had a mean practice experience of 6.9 years (SD=4.8). Most participants were of Malay ethnicity (98.6%), reflecting the local demographic composition of the Kelantan public health workforce. Only 11.8% of the participants had attended a menopause-related course, and only 2.8% reported having a personal history of receiving MHT or close family members who had experienced MHT. The sociodemographic and job characteristics of the participants are presented in Table 1.

**Table 1:** Sociodemographic and job characteristics of the participants (N=212).

Variable	n (%)	Mean (SD)
Sociodemographic characteristics		
Age		35.6 (5.3)
Sex		
Male	33 (15.6)	
Female	179 (84.4)	
Race		
Malay	209 (98.6)	
Non-Malay	3 (1.4)	
Personal history/close family members receiving MHT		
Yes	6 (2.8)	
No	206 (97.2)	
Job characteristics		
Job designation		
Medical officer	198 (93.4)	
FMS	14 (6.6)	
Place of practice Health clinic without an FMS/with a visiting FMS Health clinic with an in-house FMS	43 (20.3) 169 (79.7)	
Training/work experience in the gynaecology department Yes No	30 (14.2) 182 (85.8)	
Using CPGs as a source of information Yes No	172 (81.1) 40 (18.9)	
Years of practice		6.9 (4.8)
Attended a menopause-related course Yes No	25 (11.8) 187 (88.2)	
Estimated number of female patients aged >40 years seen per week		43.3 (33.0)

SD: standard deviation, FMS: family medicine specialist, CPG: clinical practice guideline, MHT: menopausal hormone therapy.

### Knowledge and attitude scores

Among the 212 participants, the mean knowledge score was 19.45 (SD=4.90) out of a maximum possible score of 30, with an observed range of 4–30. This corresponded to a mean percentage score of 64.8%. Conversely, the mean attitude score was 38.97 (SD=3.83) out of a maximum possible score of 50, with an observed range of 30–50.

### Knowledge of menopause management

Most participants correctly identified the clinical definition of menopause as 12 months of amenorrhoea (90.1%), hot flushes as a symptom of perimenopause (98.6%), irritability as a symptom of perimenopause (97.6%) and the role of MHT in improving quality of life among symptomatic women (99.1%).

Smaller correct-response proportions were observed for several clinical items. Only 40.6% of the participants correctly identified frequent urinary tract infections as a menopausal symptom, while 35.4% correctly recognised that follicular-stimulating hormone (FSH) testing is not routinely indicated for diagnosing menopause in women older than 45 years. The correct-response proportions were also smaller for managing isolated genitourinary syndrome of menopause (38.7%) and for correctly identifying the statement that MHT use is limited to 5 years as false (33.0%). These findings indicated variations in knowledge across different aspects of menopause management. The item-level responses are detailed in Table 2.

**Table 2:** Descriptive statistics of the items in the knowledge section of the questionnaire (N=212).

No.	Item	Answer response, n (%)			Response, n (%)			Mean (SD)
		True	False	Unsure	Correct	Incorrect		
	**Definition**						
1	The diagnosis of menopause is made after a woman has had no menses for 12 months.	191 (90.1)	15 (7.1)	6 (2.8)	191 (90.1)	21 (9.9)	0.90 (0.30)
2	Premature menopause is the cessation of ovarian function before the age of 50 years.	106 (50.0)	88 (41.5)	18 (8.5)	88 (41.5)	124 (58.5)	0.42 (0.49)
	**Symptoms**						
3	Hot flushes are a symptom of perimenopause.	209 (98.6)	1 (0.5)	2 (0.9)	209 (98.6)	3 (1.4)	0.99 (0.12)
4	Night sweats are a symptom of perimenopause.	162 (76.4)	37 (17.5)	13 (6.1)	162 (76.4)	50 (23.6)	0.76 (0.43)
5	Irritability is a symptom of perimenopause.	207 (97.6)	5 (2.4)	0 (0.0)	207 (97.6)	5 (2.4)	0.98 (0.15)
6	Painful intercourse is a symptom of perimenopause.	154 (72.6)	46 (21.7)	12 (5.7)	154 (72.6)	58 (27.4)	0.73 (0.45)
7	Frequent urinary tract infections are a symptom of perimenopause.	86 (40.6)	96 (45.3)	30 (14.2)	86 (40.6)	126 (59.4)	0.41 (0.49)
	**Risk and complications of menopause**						
8	The incidence of coronary heart disease is two to three times higher in postmenopausal women.	181 (85.4)	11 (5.2)	20 (9.4)	181 (85.4)	31 (14.6)	0.85 (0.35)
9	The risk of ischaemic stroke is reduced after 10 years of menopause.	22 (10.4)	140 (66.0)	50 (23.6)	140 (66.0)	72 (34.0)	0.66 (0.47)
	**Investigation**						
10	Medical screening for perimenopausal women includes assessment of fasting blood glucose and fasting lipid profile.	184 (86.8)	15 (7.1)	13 (6.1)	184 (86.8)	28 (13.2)	0.87 (0.34)
11	Blood test for follicular-stimulating hormone should be considered upon diagnosis of menopause in women after 45 years of age.	93 (43.9)	75 (35.4)	44 (20.8)	75 (35.4)	137 (64.6)	0.35 (0.48)
	**Indication for MHT**						
12	MHT is recommended for women whose quality of life is affected by symptoms of menopause.	210 (99.1)	0 (0.0)	2 (0.9)	210 (99.1)	2 (0.9)	0.99 (0.10)
13	MHT may prevent cardiometabolic illness, stroke and bone fractures if initiated during perimenopause.	162 (76.4)	30 (14.2)	20 (9.4)	162 (76.4)	50 (23.6)	0.76 (0.43)
14	Patients with solely genitourinary syndrome of menopause are advised to use oral MHT.	70 (33.0)	82 (38.7)	60 (28.3)	82 (38.7)	130 (61.3)	0.39 (0.49)
	**Contraindication for MHT**						
15	MHT should be offered to those who have had menopause for more than 10 years.	24 (11.3)	144 (67.9)	44 (20.8)	144 (67.9)	68 (32.1)	0.68 (0.47)
16	Liver disease is a contraindication for MHT.	130 (61.3)	19 (9.0)	63 (29.7)	130 (61.3)	82 (38.7)	0.61 (0.49)
17	Undiagnosed uterine bleeding is a contraindication for MHT.	167 (78.8)	14 (6.6)	31 (14.6)	167 (78.8)	45 (21.2)	0.79 (0.41)
18	Uncontrolled hypertension is a contraindication for MHT.	136 (64.2)	44 (20.8)	32 (15.1)	136 (64.2)	76 (35.8)	0.64 (0.48)
	**Initiation and continuation of MHT**						
19	Women with an absent uterus need oestrogen and progesterone as MHT.	75 (35.4)	91 (42.9)	46 (21.7)	91 (42.9)	121 (57.1)	0.43 (0.50)
20	Low-dose combined oral contraceptive pills can be used as MHT for perimenopausal women.	139 (65.6)	17 (8.0)	56 (26.4)	139 (65.6)	73 (34.4)	0.66 (0.48)
21	Contraception is not required within 1 year after the last menstrual period.	65 (30.7)	108 (50.9)	39 (18.4)	108 (50.9)	104 (49.1)	0.51 (0.50)
22	Menopausal women are advised to take a continuous combination of oestrogen and progesterone therapy.	86 (40.6)	81 (38.2)	45 (21.2)	86 (40.6)	126 (59.4)	0.41 (0.49)
23	Initiating MHT after the age of 60 years increases the risk of coronary heart disease and stroke.	107 (50.5)	47 (22.2)	58 (27.4)	107 (50.5)	105 (49.5)	0.50 (0.50)
24	Postmenopausal women are allowed to use MHT for 5 years only.	46 (21.7)	70 (33.0)	96 (45.3)	70 (33.0)	142 (67.0)	0.33 (0.47)
	**Side effects of MHT**						
25	Breast tenderness is a side effect of MHT.	171 (80.7)	15 (7.1)	26 (12.3)	171 (80.7)	41 (19.3)	0.81 (0.40)
26	Irregular menstrual bleeding is a side effect of MHT.	152 (71.7)	32 (15.1)	28 (13.2)	152 (71.7)	60 (28.3)	0.72 (0.45)
27	Abdominal bloating is a side effect of MHT.	143 (67.5)	25 (11.8)	44 (20.8)	143 (67.5)	69 (32.5)	0.67 (0.47)
	**Factors associated with non-response to MHT**						
28	Poor gastric absorption can cause a poor response to MHT.	141 (66.5)	17 (8.0)	54 (25.5)	141 (66.5)	71 (33.5)	0.67 (0.47)
29	Hypothyroidism can be a potential cause of non-response to MHT.	107 (50.5)	24 (11.3)	81 (38.2)	107 (50.5)	105 (49.5)	0.50 (0.50)
30	Diabetes mellitus is one of the causes of non­response to MHT.	101 (47.6)	48 (22.6)	63 (29.7)	101 (47.6)	111 (52.4)	0.48 (0.50)

SD: standard deviation, MHT: menopausal hormone therapy.

### Attitudes towards menopause management

Overall, the participants demonstrated positive attitudes towards menopause management. Most agreed that training in menopause management is essential (76.4% strongly agreed) and that MHT should be offered to eligible women without contraindications (84.9% agreed or strongly agreed).

The largest proportion of the participants (43.4%) selected a neutral response regarding whether they were prepared to manage menopausal women. The complete response distributions for the individual attitude items are shown in Table 3.

**Table 3:** Descriptive statistics of the items in the attitude section of the questionnaire (N=212).

No.	Item	Answer response, n (%)					Mean (SD)
		Strongly disagree	Disagree	Neutral	Agree	Strongly agree	
1	I think it is essential to be trained in menopause management.	0 (0.0)	0 (0.0)	2 (0.9)	48 (22.6)	162 (76.4)	4.75 (0.45)
2	I am prepared to manage menopausal women.	6 (2.8)	36 (17.0)	92 (43.4)	51 (24.1)	27 (12.7)	3.27 (0.98)
3	I think MHT should be offered to women with menopausal symptoms who do not have contraindications.	0 (0.0)	2 (0.9)	30 (14.2)	110 (51.9)	70 (33.0)	4.17 (0.69)
4	I will recommend MHT to my family and friends who are indicated for MHT.	0 (0.0)	1 (0.5)	40 (18.9)	111 (52.4)	60 (28.3)	4.08 (0.70)
5	I think a trained health practitioner should prescribe MHT.	0 (0.0)	0 (0.0)	24 (11.3)	110 (51.9)	78 (36.8)	4.25 (0.65)
6	I think MHT has a limited role in improving menopausal women’s quality of life.	22 (10.4)	98 (46.2)	41 (19.3)	40 (18.9)	11 (5.2)	3.38 (1.07)
7	I should screen menopausal women for osteoporosis.	1 (0.5)	3 (1.4)	12 (5.7)	117 (55.2)	79 (37.3)	4.27 (0.67)
8	I think MHT has more side effects than benefits.	7 (3.3)	96 (45.3)	88 (41.5)	18 (8.5)	3 (1.4)	3.41 (0.75)
9	I think MHT should be made available in health clinics.	0 (0.0)	9 (4.2)	41 (19.3)	125 (59.0)	37 (17.5)	3.90 (0.73)
10	I think MHT is cost-effective.	2 (0.9)	8 (3.8)	106 (50.0)	77 (36.3)	19 (9.0)	3.49 (0.75)
		Mean total score					38.97 (3.83)

SD: standard deviation, MHT: menopausal hormone therapy

### Practices regarding menopause management

Among activities relevant to the national CPGs, 46.7% of the participants reported that they sometimes discussed menopause with patients in the perimenopausal age group, with 42.4% reported doing so rarely or never. More than half indicated never prescribing or referring women with menopausal symptoms for MHT (52.8%), while 53.8% reported never prescribing or referring eligible women for MHT for the prevention or treatment of osteoporosis. Lifestyle counselling was among the more frequently noted activities, with only 5.7% reporting that they never provided such advice. In contrast, 33.5% indicated never advising women receiving MHT to attend annual consultations, and 42.9% reported never counselling patients about the possible adverse effects of MHT. More than half never advised patients regarding lifelong MHT use (55.7%). The item-level practice responses are detailed in Table 4.

**Table 4:** Descriptive statistics of the items in the practice section of the questionnaire (N=212).

No.	Item	Answer response, n (%)					Mean (SD)
		Never	Rarely	Sometimes	Always	Often	
1	I discuss menopause with my patients who are in the perimenopausal age group.	14 (6.6)	76 (35.8)	99 (46.7)	21 (9.9)	2 (0.9)	2.63 (0.79)
2	I prescribe or refer women with menopausal symptoms for MHT.	112 (52.8)	55 (25.9)	34 (16.0)	10 (4.7)	1 (0.5)	1.74 (0.93)
3	I prescribe or refer women for MHT to prevent cardiovascular diseases.	125 (59.0)	50 (23.6)	27 (12.7)	9 (4.2)	1 (0.5)	1.64 (0.90)
4	I prescribe or refer women for MHT to prevent or treat osteoporosis.	114 (53.8)	54 (25.5)	32 (15.1)	11 (5.2)	1 (0.5)	1.73 (0.93)
5	I prescribe or refer women for MHT to prevent or treat cognitive symptoms.	128 (60.4)	46 (21.7)	31 (14.6)	6 (2.8)	1 (0.5)	1.61 (0.87)
6	I advise my patients on lifestyle changes as part of menopause management.	12 (5.7)	31 (14.6)	80 (37.7)	71 (33.5)	18 (8.5)	3.25 (1.00)
7	I advise women with menopausal symptoms to use herbal products such as black cohosh or evening primrose oil.	128 (60.4)	40 (18.9)	37 (17.5)	5 (2.4)	2 (0.9)	1.65 (0.91)
8	I advise women on MHT on annual consultations.	71 (33.5)	39 (18.4)	49 (23.1)	43 (20.3)	10 (4.7)	2.44 (1.27)
9	I advise patients regarding the side effects of MHT.	91 (42.9)	51 (24.1)	44 (20.8)	22 (10.4)	4 (1.9)	2.04 (1.11)
10	I advise patients on life-long MHT.	118 (55.7)	44 (20.8)	38 (17.9)	10 (4.7)	2 (0.9)	1.75 (0.97)

SD: standard deviation, MHT: menopausal hormone therapy

### Factors associated with the knowledge scores

In the SLR analysis, the knowledge scores were significantly higher among the older participants, participants with an FMS designation, participants with longer practice duration, participants with prior gynaecology experience, participants who attended menopause management training and participants who used CPGs as an information source.

In the MLR model, three factors remained significant independent predictors of higher knowledge scores. Age was positively associated with knowledge, with each additional year increasing the score by 0.21 points (adjusted β=0.21; 95% CI=0.08–0.34; P=0.002). FMS designation was also a significant predictor, with the specialists scoring 3.06 points higher than the medical officers (adjusted β=3.06; 95% CI=0.26–5.81; P=0.032). Additionally, use of CPGs as a source of information was associated with a 1.91-point increase in the knowledge score (adjusted β=1.91; 95% CI=0.30–3.52; P=0.021). The other sociodemographic and job-related variables were not significantly associated with the knowledge scores in the adjusted model. The detailed regression results are presented in Table 5.

**Table 5:** Factors associated with knowledge on menopause management among the participants (N=212).

Item	Simple linear regression			Multiple linear regression			
		Crude b^a^	95% CI	P	Adjusted b^b^	95% CI	t	P
**Sociodemographic characteristics**							
Age (year)	0.26	0.14, 0.38	**<0.001**	0.21	0.08, 0.34	4.126	0.002
Sex Male Female	Ref. 0.61	-1.23, 2.44	0.514				
Race Malay Non-Malay	Ref. 2.25	-3.38, 7.87	0.432				
Personal history/close family members receiving MHT No Yes	Ref. -0.47	-4.48, 3.55	0.819				
**Job characteristics**							
Job designation Medical officer FMS	Ref. 5.17	2.59, 7.76	**<0.001**	3.06	0.26, 5.81	2.155	0.032
Place of practice Health clinic without an FMS/with a visiting FMS Health clinic with an in-house FMS	Ref. -0.04	-1.70, 1.61	0.958				
Training/work experience in the gynaecology department No Yes	Ref. 2.5	0.62, 4.38	**0.009**				
Using CPGs as a source of information No Yes	Ref. 1.91	0.23, 3.60	**0.026**	1.91	0.30, 3.52	2.335	0.021
Years of practice	0.23	0.09, 0.36	**0.001**				
Attended a menopause-related course No Yes	Ref. 2.8	0.77, 4.83	**0.007**				
Estimated number of female patients aged ≥40 years seen per week	0	-0.02, 0.02	0.933				

a Crude regression coefficient

b Adjusted regression coefficient (R^2^ = 0.114). VIF values for the final model: 1.23 for age, 1.24 for job designation and 1.02 for use of CPGs as a source of information, indicating no multicollinearity.

FMS: family medicine specialist, CI: confidence interval, SD: standard deviation, MHT: menopausal hormone therapy, CPG: clinical practice guideline

## Discussion

### Principal findings

This study provides an overview of knowledge, attitudes and self-reported activities regarding menopause management among PCPs in Kelantan. Overall, the participants demonstrated good recognition of several core menopause-related concepts and generally favourable attitudes towards menopause care. However, smaller correct-response proportions were observed for several clinical aspects of diagnosis and MHT. Lifestyle counselling was commonly reported, whereas several MHT- related prescribing, referral, monitoring and counselling activities were indicated less frequently. Older age, FMS designation and use of the Malaysian CPGs as an information source were independently associated with higher knowledge scores.

### Knowledge of menopause management

The participants demonstrated large correct-response proportions for several foundational items, including the definition ofmenopause as 12 months of amenorrhoea (90.1%) and the recognition of common symptoms such as hot flushes (98.6%) and irritability (97.6%). This pattern is consistent with the findings of Al-Huthali et al. in Saudi Arabia, where 95.8% and 96.6% of family medicine residents easily recognised vasomotor symptoms and vaginal dryness, respectively.^11^

However, smaller correct-response proportions were noted for several clinical items. Only 40.6% of the participants recognised frequent urinary tract infections as a possible genitourinary manifestation associated with menopause. Although knowledge does not necessarily reflect clinical practice, this finding may have clinical implications because primary care settings are often the first point of contact for women presenting with urinary symptoms, and failure to recognise menopause-related causes may delay appropriate assessment and management.^5^

Only 35.4% correctly recognised that FSH testing is not routinely indicated for diagnosing menopause in otherwise healthy women older than 45 years. This result demonstrates limited knowledge of the diagnostic recommendation but does not establish actual testing behaviour. Similar deficiencies in technical knowledge have been reported internationally. In mainland China, Lin et al. found that 41.8% ofhealthcare professionals incorrectly considered an elevated FSH level mandatory for diagnosing natural menopause, while Kling et al. showed that 16.4% of postgraduate medical residents in the United States incorrectly identified FSH testing as a diagnostic requirement.^12,13^

Knowledge of MHT regimen selection also varied. Only 42.9% of the participants recognised that women without a uterus do not require progesterone therapy as a part of MHT; 21.7% were unsure, while 35.4% answered incorrectly. These responses suggest uncertainty about regimen selection, although actual prescribing behaviour was not assessed. Unnecessary administration of progestogen may expose women to risks without providing the intended endometrial benefit.

Only half of the participants correctly identified that initiating MHT after the age of 60 years is associated with an increased cardiovascular risk. This finding suggests incomplete understanding of the ‘timing hypothesis’, which proposes that the balance of the benefits and risks of MHT varies according to a woman’s age and time since menopause. Current evidence indicates that women who initiate MHT before the age of 60 years or within 10 years of menopause generally have a more favourable benefit—risk profile than those initiating treatment later.^14,15^ Although the relationship between this knowledge gap and clinical practice could not be determined, improving the understanding of contemporary evidence regarding MHT may support more informed counselling and shared decision-making.

### Attitudes towards menopause management

The participants generally expressed favourable attitudes towards menopause management, including support for training and for offering MHT to eligible symptomatic women. However, the responses regarding self-perceived preparedness were less definitive, with the largest proportion (43.4%) selecting a neutral response. This showed that although the participants recognised the importance of menopause care, many were uncertain about their readiness to manage menopausal women in routine clinical practice.

The responses to the MHT safety item were generally favourable. Only 9.9% of the participants agreed or strongly agreed that MHT has more adverse effects than benefits, whereas 48.6% disagreed or strongly disagreed. However, 41.5% selected a neutral response, indicating that uncertainty about the balance of MHT risks and benefits remained common despite the small proportion expressing an unfavourable view.

Reluctance to prescribe MHT may be influenced by concerns regarding its risks and adverse effects. Low et al. reported that 79.8% of primary care doctors identified patients’ fear of breast cancer as a barrier to MHT use, while 67.3% expressed concerns about MHT side effects.^7^ However, the present study did not directly assess specific barriers such as concerns about breast cancer, clinician- related safety concerns or system-level obstacles.

### Self-reported menopause management practices

Lifestyle counselling was among the most frequently reported menopause management activities. This finding is consistent with that of Low et al., who showed that 98.4% of Malaysian primary care doctors recommended lifestyle modification as their main intervention.^16^ Although lifestyle modification is an important component of menopause care, it may be insufficient for managing severe vasomotor symptoms or addressing substantial osteoporosis risks.^14^

More than half of the participants (52.8%) reported never prescribing or referring women with menopausal symptoms for MHT. Prescribing or referring patients for MHT for osteoporosis prevention or treatment was also noted infrequently. Furthermore, annual review and counselling regarding possible adverse effects were less frequently described.

A cross-sectional study among primary care doctors in public health clinics in Selangor and Kuala Lumpur similarly reported frequent discussions of MHT but limited direct prescriptions: Only 0.9% prescribed MHT, whereas 66.0% referred patients to tertiary care.^16^ Direct comparisons with the present study should be made cautiously because prescription and referral were combined in a single questionnaire item, whereas the Klang Valley study reported these activities separately.

Taken together, the findings suggest that MHT-related management may be infrequently incorporated into routine primary care in both settings. However, the present study did not assess patient eligibility, contraindications, service availability, referral pathways or the clinical rationale underlying management decisions. The differences between the studies may also reflect variations in questionnaire designs, sampling methods, healthcare settings and treatment availabilities. Comparable differences between favourable attitudes and reported prescription practices have also been described internationally. In Saudi Arabia, Qutob et al. showed that although 60% of physicians agreed that MHT should be offered, prescription was influenced by perceived barriers and patient preferences.^17^ Similarly, Y;ganeh et al. found that although 91% of Australian health professionals were willing to offer MHT to symptomatic women, reported practices varied across medical specialties.^18^ These studies provide relevant context, but the barriers identified in those settings were not directly assessed in the present study.

### Factors associated with knowledge

Older age, FMS designation and use of the Malaysian CPGs as a source of information were independently associated with higher knowledge scores on menopause management. The association between older age and knowledge may reflect greater cumulative clinical experience and exposure to menopause management over time, although age may also represent other unmeasured factors such as seniority or continuing professional development. Similarly, the FMSs had higher knowledge scores than the medical officers. This difference may reflect variations in postgraduate training, clinical experience or professional responsibilities; however, these factors were not directly examined in the present study. Moreover, the FMSs represented only 6.6% of the sample, and the findings related to specialist designation should therefore be interpreted cautiously. The predominance of medical officers in the sample (93.4%) suggests that future educational initiatives should be accessible and relevant to this group.

The association between CPG use and a higher knowledge score suggests that engagement with clinical guidelines may support knowledge acquisition, although the cross-sectional design does not establish a causal relationship. In this study, 81.1% of the participants reported using CPGs, whereas Low et al. found that 39.7% of primary care doctors in an urban Malaysian sample were unaware of such guidelines.^16^ The differences between these findings may be related to variations in settings, sampling methods or study periods.

Standardised educational frameworks may be strengthened using the Malaysian CPGs as the core content for a structured menopause-related training curriculum. The guidelines could provide the foundation for teaching menopause assessment, diagnosis, treatment selection, contraindications, monitoring and referral, while practical components such as case-based discussions, prescription algorithms and decision-support tools may help translate recommendations into clinical practice.

### Clinical and health system implications

The observed knowledge gaps and variations in self-reported practices may affect menopause care in Malaysian primary care settings. Frequent vasomotor symptoms are associated with adverse cardiovascular risk profiles and subsequent cardiovascular diseases.^19^ Declining oestrogen levels during menopause also accelerate bone loss and increase fracture risk.^14^ Primary care therefore offers an important opportunity window for early risk assessment, counselling, preventive intervention and referral.

Malaysia is expected to face a substantial rise in osteoporotic hip fractures.^4^ In this study, prescription of or referral for MHT for osteoporosis was infrequently reported, although patient-level factors and clinical reasons were not assessed. Therefore, this finding does not establish inappropriate withholding of MHT. Earlier osteoporosis risk assessment remains important because the condition may go undiagnosed until a fragility fracture occurs.^20^

These findings support strengthening primary care capacity for individualised menopause management through education on symptom assessment, cardiovascular and bone health evaluation, treatment options, shared decision-making and referral pathways.

### Strengths and limitations

The strength of this study lies in its assessment of PCPs’ knowledge, attitudes and self-reported practices regarding menopause management in Kelantan, addressing a geographical gap in the Malaysian literature, which has previously been limited to the Klang Valley. The questionnaire was adapted from national CPGs, with item-level reporting enabling specific knowledge gaps and patterns of reported practice to be identified.

Several limitations should also be acknowledged. First, the cross-sectional design prevents causal inference, and self-reported practices may be affected by recall and social desirability bias. Second, the use of convenience sampling may have introduced selection bias. Recruitment through professional WhatsApp and FMS networks may have disproportionately attracted providers who were more academically engaged or more interested in menopause care. As the sampling denominator was unknown, response rates could not be calculated, and information on non-responders was unavailable, precluding the assessment of potential non-response bias. Therefore, the findings should be interpreted with caution and may not be fully generalisable to all PCPs in Kelantan. Third, the questionnaire did not directly assess prescription barriers, clinical confidence, institutional factors or reasons for prescription and referral decisions. Finally, the small number of FMS participants (6.6%) limits the precision and generalisability of the specialist-specific findings, although designation was included in the regression model. The differences between the pilot and target clinical settings may also limit the transferability of the pilot findings to all public health clinic contexts.

## Conclusion

The PCPs in Kelantan demonstrated knowledge of several menopause-related concepts and generally favourable attitudes. However, gaps were identified in specific technical knowledge areas, and several MHT-related prescription, referral and counselling activities were infrequently reported. The study did not determine the reasons underlying these patterns. Future research should directly assess clinician-, patient- and health system-related factors and evaluate appropriate educational and service-level interventions.

## Data sharing statement

The data generated in this study will be made available upon request to the corresponding author.

**How does this paper make a difference in general practice?**

- The study identifies specific knowledge gaps and uncertainty in self-reported preparedness regarding menopause management in Malaysian primary care.
- It identifies a discrepancy between favourable attitudes and infrequent self-reported menopausal hormone therapy-related practices.
- It provides a rationale for directly assessing barriers and evaluating guideline-based, practice-focused interventions for medical officers.
- It highlights the association between use of the Malaysian clinical practice guidelines and higher knowledge scores.

## References

[1] Dhillon HK, Singh HJ, Shuib R, Hamid AM, Mohd Zaki Nik Mahmood N (2006). Prevalence of menopausal symptoms in women in Kelantan, Malaysia.. Maturitas..

[2] Ismael NN (1994). A study on the menopause in Malaysia.. Maturitas..

[3] Abdullah B, Moize B, Ismail BA, Zamri M, Mohd Nasir NF (2017). Prevalence of menopausal symptoms, its effect to quality of life among Malaysian women and their treatment seeking behaviour.. Med J Malaysia..

[4] Cheung CL, Ang SB, Chadha M (2018). An updated hip fracture projection in Asia: The Asian Federation of Osteoporosis Societies study.. Osteoporos Sarcopenia..

[5] Obstetrical and Gynaecological Society of Malaysia, Malaysian Menopause Society (2022). Clinical Practice Guidelines: Management of Menopause in Malaysia.

[6] Alwi SA, Rubiah ZS, Lee PY, Mallika PS, Haizal MN (2010). Experience of hormone replacement therapy among women of Sarawak, Malaysia.. Climacteric..

[7] Low TL, Cheong AT, Devaraj NK, Ismail R (2024). Prevalence of offering menopause hormone therapy among primary care doctors and its associated factors: A cross-sectional study.. PLoS One..

[8] Hasson D, Arnetz BB (2005). Validation and findings comparing VAS vs. Likert scales for psychosocial measurements.. Int Electron J Health Educ..

[9] Arifin WN Sample size calculator. https://wnarifin.github.io/ssc/ss1mean.html.

[10] Hosmer DW, Lemeshow S, Sturdivant RX (2013). Applied Logistic Regression.

[11] Al-Huthali M, Bahattab E, Alqurashi J Knowledge, attitude and practice towards management of postmenopausal symptoms in primary health care among family medicine residents in Makkah City, 2021: A cross-sectional study..

[12] Kling JM, MacLaughlin KL, Schnatz PF (2019). Menopause Management Knowledge in Postgraduate Family Medicine, Internal Medicine, and Obstetrics and Gynecology Residents: A Cross-Sectional Survey.. Mayo Clin Proc..

[13] Lin L, Feng P, Yu Q (2020). Attitude and knowledge for menopause management among health professionals in mainland China.. Climacteric..

[14] (2022). The 2022 hormone therapy position statement of The North American Menopause Society.. Menopause..

[15] Hodis HN, Mack WJ, Henderson VW (2016). Vascular effects of early versus late postmenopausal treatment with estradiol.. N Engl J Med..

[16] Low TL, Cheong AT, Devaraj NK, Ismail R (2025). Primary care doctors’ practices in managing menopausal symptoms and views on patient clinical conditions influencing their decision to offer menopause hormone therapy: A cross-sectional study.. Malays Fam Physician..

[17] Qutob RA, Alaryni A, Alsolamy EN (2024). Attitude, practices, and barriers to menopausal hormone therapy among physicians in Saudi Arabia.. Cureus..

[18] Yeganeh L, Boyle J, Teede H, Vincent A (2017). Knowledge and attitudes of health professionals regarding menopausal hormone therapies.. Climacteric..

[19] El Khoudary SR, Aggarwal B, Beckie TM (2020). Menopause transition and cardiovascular disease risk: Implications for timing of early prevention: A scientific statement from the American Heart Association.. Circulation..

[20] MOH (2022). Clinical Practice Guidelines: Management of Osteoporosis.

